# Composition Influence on Pulmonary Delivery of Rifampicin Liposomes

**DOI:** 10.3390/pharmaceutics4040590

**Published:** 2012-11-27

**Authors:** Maria Letizia Manca, Chiara Sinico, Anna Maria Maccioni, Octavio Diez, Anna Maria Fadda, Maria Manconi

**Affiliations:** 1 Department of Environmental and Life Science, University of Cagliari, Cagliari 09124, Italy; Email: mlmanca@unica.it (M.L.M.); sinico@unica.it (C.S.); macciom@unica.it (A.M.M.); mfadda@unica.it (A.M.F.); 2 Department of Pharmacy and Pharmaceutical Technology, University of Valencia, Valencia 46100-Burjassot, Spain; Email: octavio.diez@uv.es

**Keywords:** liposomes, rifampicin, cholesterol, oleic acid, rheology, pulmonary delivery, aerosol, cell viability, cellular uptake

## Abstract

The effects of lipid concentration and composition on the physicochemical properties, aerosol performance and *in vitro* toxicity activity of several rifampicin-loaded liposomes were investigated. To this purpose, six liposome formulations containing different amounts of soy phosphatidylcholine and hydrogenated soy phosphatidylcholine, with and without cholesterol and oleic acid, were prepared and fully characterized. Uni- or oligo-lamellar, small (~100 nm), negatively charged (~60 mV) vesicles were obtained. Lipid composition affected aerosol delivery features of liposomal rifampicin; in particular, the highest phospholipid concentration led to a better packing of the vesicular bilayers with a consequent higher nebulization stability. The retention of drug in nebulized vesicles (NER%) was higher for oleic acid containing vesicles (55% ± 1.4%) than for the other samples (~47%). A549 cells were used to evaluate intracellular drug uptake and *in vitro* toxicity activity of rifampicin-loaded liposomes in comparison with the free drug. Cell toxicity was more evident when oleic acid containing liposomes were used.

## 1. Introduction

Rifampicin is an effective antibiotic used in anti-tuberculosis therapy, but its treatment involves prolonged oral administration of high systemic doses over a period of 4–10 months. The long duration of anti-tubercular chemotherapy is related with various systemic side effects and poor patient compliance. The treatment of chronic lung infection, especially for maintenance treatment, opens a door of opportunity for inhaled antibiotics, as the lungs are directly targeted. Although particle deposition at the site of interest is crucial in determining the therapeutic efficacy of inhaled therapeutics, mucociliary clearance may considerably limit the residence time of the deposited drug and, subsequently, the uptake by the target cells. Moreover, the nature and extent of drug interactions with lung lining fluid, airway macrophages and lung epithelial cells affect its permanence *in situ* and, therefore, the duration of the effect.

To overcome these drawbacks, targeted delivery of drugs to the lungs through various drug delivery systems, such as polymeric micro-nanoparticles, liposomes, niosomes, and dendrimers represents a promising strategy. In fact, nano- and micro-carrier technology plays an important role in providing new drug delivery systems that can improve both drug solubility and stability against metabolism and degradation [1,2,3]. Moreover, they allow a relatively uniform dose distribution among the alveoli, delay drug residence time in the tissue and control its release [4,5,6,7,8,9,10,11]. Sustained drug delivery to the respiratory tract provides extended duration of action, a reduction in the therapeutic dose of drugs, improved management of therapy, improved patient compliance and a reduction of the adverse effects of highly toxic drugs [12,13,14]. In addition, inhaled microparticles are recognized as alien by alveorar macrophages, which phagocytose them and initiate innate immune responses. Geiser *et al.* demonstrated an increase in the numbers of airway/lung macrophages by approximately three times immediately after inhalation of polystyrene microparticles by hamsters [15,16,17,18]. Among the several carriers used for pulmonary application, liposomes are one of the most extensively investigated systems due to their high biodegradability and biocompatibility, and their capability to facilitate intracellular delivery and prolong the retention time of entrapped agents inside the cell. Moreover, they are easily biofunctionalizable, being the liposomal surface modifiable by wrapping in biocompatible materials or conjugation with specific ligands, which increase the targeting efficiency to specific tissues or organs. Indeed, advanced research in liposome technology has allowed achievement of suitable formulations able to improve drug bioavailability in the lung tissue. Vyas *et al.* demonstrated that encapsulation of anti-tubercular drugs in the liposomes, modification of the liposomal surface by negative charge and macrophage-specific ligands, and deposition to respiratory tract via aerosolization, improved the chemotherapy against pulmonary tuberculosis [19]. Chono *et al.* prepared mannosylated ciprofloxacin-liposomes with particle size ~1000 nm and found that the targeting efficiency of ciprofloxacin to rat alveolar macrophages following pulmonary administration of mannosylated CPFX-liposomes was significantly greater than that of ciprofloxacin incorporated into unmodified liposomes [20]. Liposomal ciprofloxacin from Aradigm corp. is currently in Phase 2 programs for respiratory infections associated to cystic fibrosis and bronchiectasis.

In our previous studies, liposomes and coated liposomes were developed to improve rifampicin pulmonary delivery [21,22,23]. In particular, we investigated applicability of rifampicin containing chitosan-coated liposomes as a carrier for delivery of drugs to the lungs by nebulization. We found that mucoadhesive properties of coated liposomes were substantially better (compared with non-coated ones), whereas the toxicity of liposomal rifampicin towards A549 epithelial cells was lower compared with the free drug. 

In the present work, in an attempt to improve the liposome capability to deliver rifampicin to the pulmonary tissue, the influence of vesicle composition on their physico-chemical properties, aerosol performance and cell interaction ability of the bilayered vesicles was evaluated. To this purpose, rifampicin was entrapped in liposomes prepared using a binary mixture of soy phosphatidylcholine (P50) and hydrogenated soy phosphatidylcholine (P90H) as reported in previous studies [22,23]. The association of P90H, characterized by a high transition temperature (*T*m = 52 °C), and P50, with low *T*m (~ −10°C), was used in two different concentrations (30 and 60 mg/mL). Moreover, the composition was varied by adding cholesterol alone or cholesterol and oleic acid to evaluate the influence of all the components on vesicle physico-chemical properties and, in particular, on their capability to incorporate stably high amounts of RFP.

## 2. Results and Discussion

### 2.1. Characterization of Liposomes.

All formulations were prepared using a binary mixture of hydrogenated soy phosphatidylcholine (P90H, transition temperature 52 °C) and soy phosphatidylcholine (P50, transition temperature ~ −10 °C), which allowed us to obtain stable systems capable of incorporating high amounts of RFP. To optimize the liposome performance as aerosol carriers, phospholipid mixtures were used at two lipid concentrations (30 and 60 mg/mL). Moreover, these two basic formulations were modified by adding cholesterol alone or cholesterol and oleic acid. The first one is able to modify and stabilize the fluidity of the bilayer [24], whereas the former regulates the membrane packing and confers a negative charge to vesicle surfaces [25]. Composition of the six different liposomal formulations is reported in Table 1.

**Table 1:** Composition of vesicular dispersions.

	RFP	P90H	P50	Chol	OA
	mg/mL	mg/mL	mg/mL	mg/mL	mg/mL
PC30	10	15	15	--	--
PC30 Chol	10	15	15	10	--
PC30 Chol OA	10	15	15	10	3
PC60	10	30	30	--	--
PC60 Chol	10	30	30	10	--
PC60 Chol OA	10	30	30	10	6

**Figure 1:**
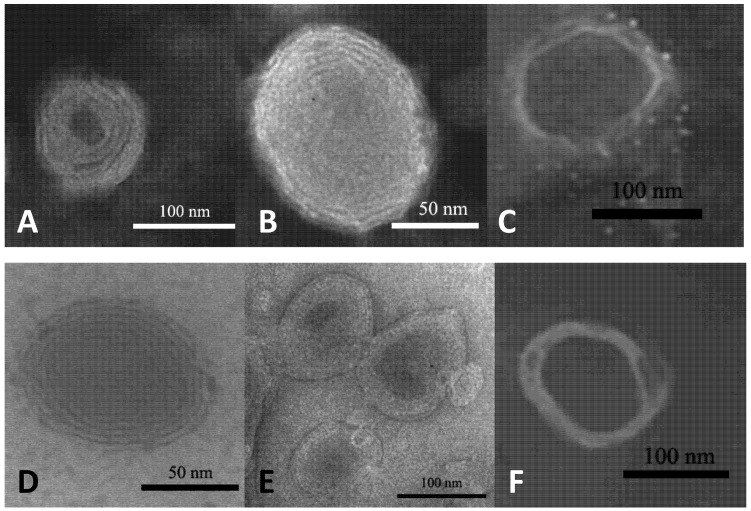
Negative electron transmission micrographs of rifampicin-loaded liposomes prepared with 30 or 60 mg/mL of phosphatidylcholine (**A**) PC30; (**B**) PC30 Chol; (**C**) PC30 Chol OA; (**D**) PC60; (**E**) PC60 Chol; (**F**) PC60 Chol OA.

TEM analyses confirmed vesicle formation and showed the presence of uni- or oligo-lamellar vesicles depending on sample composition. In fact, the presence of oleic acid in the bilayer decreased the lamellarity, and vesicles became unilamellar (Figure 1).

**Figure 2:**
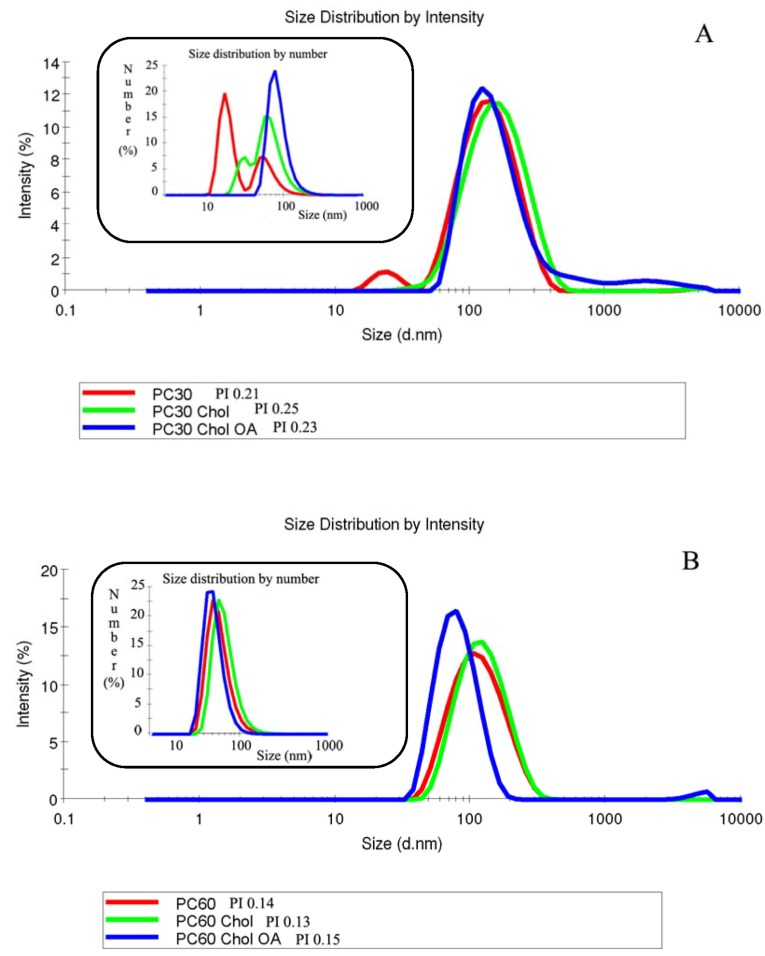
Size distribution curves of PC30 (**A**) and PC60 (**B**) samples as a function of intensity and number of vesicles, obtained from dynamic light scattering.

Non-dialyzed and dialyzed liposomes were characterized in terms of structure, size distribution, surface charge and entrapment efficiency (Table 2). Results have shown that sample composition affected vesicle mean size and distribution. There are several methods to evaluate the polydispersity index of colloidal dispersions, such as TEM analysis [26], but the most used is dynamic light scattering that permits one to estimate size distribution and relate the polydispersity index as a function of intensity and the number of particles. Figure 2A shows the size distribution of PC30 formulations measured with dynamic light scattering. As can be seen, especially from the insert showing size distribution as a function of vesicle number, all the three formulations with a PI > 0.2 were not homogeneously dispersed, but characterized by the presence of two populations. On the contrary, panel 2B shows that PC60 liposomes (PI < 0.2) are homogeneously dispersed and formed by only one population with the same mean diameter. 

The increase of phospholipid concentration (from PC30 to PC60) did not influence the vesicle size, but led to a reduction of the standard deviation and polydispersity index of the samples. Therefore, the three formulations more concentrated in phospholipids (PC60) were more homogeneous than the corresponding PC30, as confirmed by their lower PI value. The addition of cholesterol and oleic acid in PC30 liposomes increased the vesicle mean diameter that, on the contrary, was reduced in PC60. 

The mean diameter of all dialyzed formulations remained almost unchanged (*p* > 0.05) after the purification procedure, thus indicating a good stability of formulations [27]. The zeta potential of all formulations was highly negative as a consequence of their composition. The primary components of P50 and P90H are phosphatidylcholine and hydrogenated phosphatidylcholine, respectively. These compounds are characterized by a net negative charge, thus for this reason, vesicular dispersions were negatively charged. Moreover, surface charge did not increase by increasing phospholipid concentration, probably because there is not a variation of the charge density. 

A slight increase of the negative charge occurs in the presence of oleic acid, specifically added to further stabilize the vesicle dispersions, from (−50) ± 2.8 to (−67) ± 3.3 [28].

Rifampicin incorporation efficiency (E%) was higher for the liposomes most concentrated in phospholipids (PC60) than for the corresponding PC30, as found previously [23]. Previous studies have shown that RFP is inserted into the liposomal bilayers and its bulky size is responsible for the increased mean diameter of the vesicles. Moreover, it was also found that RFP incorporation efficiency can be reduced when cholesterol is included in the formulation as a consequence of RFP displacement by cholesterol. Furthermore, addition of cholesterol led to increased vesicle size [21]. Therefore, the contemporary presence of bulky molecules, such as cholesterol and RFP, has an effect on the RFP incorporation efficiency and liposomal size that is correlated to the phospholipid concentration. Phospholipids in PC30 are not sufficiently concentrated to guarantee the optimal packing of the bilayer in the presence of RFP and cholesterol, and thus, the result is an increased vesicle size. The further addition of another component, oleic acid, further enlarges the liposomes. On the contrary, a higher concentration of both unsaturated and saturated phospholipids can allow all the associated components to better pack in the liposomal bilayer structure, with a consequent vesicle size reduction and an improved drug incorporation efficiency.

**Table 2:** Mean size, polydispersity index (PI), zeta potential (ZP) and entrapment efficiency (E%) of rifampicin-liposomes.

	Before dialysis			After dialysis			
	Size (nm) ± SD	PI	ZP (mV) ± SD	Size (nm) ± SD	PI	ZP (mV) ± SD	E (%) ± SD
PC30	106 ± 11	0.21	−50 ± 2.8	101 ± 12	0.20	−58 ± 0.4	67 ± 4
PC30 Chol	128 ± 8	0.25	−57 ± 3.1	118 ± 8	0.25	−63 ± 2.9	64 ± 6
PC30 Chol OA	143 ± 15	0.23	−67 ± 3.3	153 ± 9	0.23	−67 ± 3.7	60 ± 2
PC60	104 ± 3	0.14	−54 ± 3.8	102±2	0.15	−57 ± 1.3	76 ± 4
PC60 Chol	112 ± 4	0.13	−57 ± 6.6	108 ± 3	0.17	−62 ± 1.2	74 ± 4
PC60 Chol OA	79 ± 6	0.15	−63 ± 7.4	85 ± 6	0.15	−69 ± 3.3	69 ± 2

### 2.2. Rheological Studies

**Figure 3:**
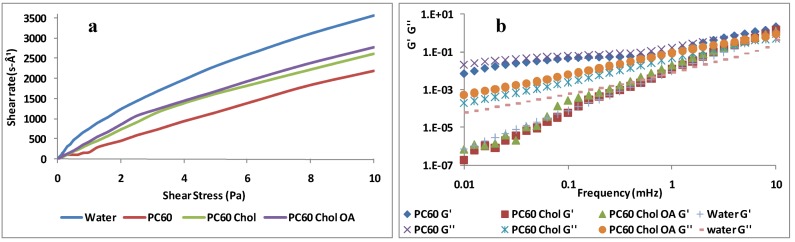
(**a**) Shear rate against shear stress for vesicles compared with water; (**b**) Frequency sweep spectra of vesicles compared with water: storage (G0) and loss (G00) moduli against frequency are shown.

Rheological analyses provide important information about the structure of the colloidal systems. Many apparently homogeneous systems, actually, consist of complex dispersions of different phases. These systems in relaxed conditions show characteristics that may change when subjected to an external stress. In this work, we evaluated the rheological behavior of rifampicin loaded liposomes. Viscometry analyses showed a Newtonian behavior for all tested formulations, where the viscosity was independent of the applied shear stress (Figure 3a) [29]. Figure 2 reports the plots of three PC60 samples; as shown, no statistical differences were observed between PC30 and PC60 samples. The sample viscosity was always higher than that of water (~1 mPa) due to the hydrodynamic volume fraction of lamellar structures [30]. PC60 liposomes showed the highest viscosity (4.19 ± 0.0005 mPa); with the addition of cholesterol, the viscosity decreased slightly (3.23 ± 0.0004 mPa), then, a further reduction in viscosity was found with the simultaneous addition of oleic acid and cholesterol (2.23 ± 0.0003). The viscosity reduction was due to the decrease of vesicle size in these dispersions. Oscillating rheological measurements (frequency sweep) were carried out in order to measure the elastic (G') and the viscous (G'') component of the systems (Figure 3b). The viscous modulus of liposomes, as well as that of water, was higher than the elastic modulus, and their values were only slightly higher than that of water. These results demonstrated that the liposome dispersions are diluted unstructured lamellar systems characterized by weak interactions between vesicles that behave as ideal Newtonian fluids, which flow following application of a stress and withstand without breaking. Vesicle stability under stress ensures a good resistance even during the aerosol flow.

### 2.3. Nebulization Studies of Liposomes

The influence of sample composition on nebulization was evaluated. Nebulizers generally operate to dryness, the point at which no further useful aerosol is generated, even if some residual fluid always remains in the nebulizer reservoir. Therefore, the total mass output, which represents the percentage of the average delivered dose, is usually calculated. The total mass output of tested samples was always less than 100%. In particular samples, PC60, PC60 Chol and PC60 Chol OA were characterized by a total mass output of 54%, 66% and 81%, respectively, whereas PC30, PC30 Chol and PC 30 Chol OA showed a total mass output of 45%, 56% and 62%, respectively. Relevant studies have shown that liposome nebulization is affected by the viscosity of the liposome dispersion, thus explaining the falls in the total mass output for PC60 or PC30 liposomes characterized by a higher viscosity value than corresponding vesicles also containing cholesterol or cholesterol and oleic acid [22,31,32].

The Nebulization Efficiency (NE%) (amount of drug nebulized compared to that inserted in the nebulizer) was evaluated in all the three stages of a home-made glass impinger [21]. This device can give information on the possible lung deposition, although it cannot provide details on the aerodynamic diameter of the particles. For all tested formulations, the highest amount of drug deposited on the impinger first stage, while drug deposition was low in stages 2 and 3; never higher than 15%. Fine particles dose (FPD) and fine particle fraction (FPF) are generally defined as the amount and the percentage of drug depositing, respectively, in the deep lung and alveolar region. The three stages of this impinger device can mimic the whole human respiratory system and, therefore, FPF and FPD were evaluated in the last two stages that represent the lower airway region [23,33]. 

PC60 Chol OA showed the highest FPD and FPF: 1685 ± 35 µg and 17% ± 0.6%, respectively (Table 3). Results show that the combination of PC, Chol and OA always improved FPF in comparison to the other liposomal formulations. The retention of RFP in nebulized vesicles (NER%) was also evaluated. It was not possible to measure this parameter in each stage of the impinger, and for this reason, at the end of the experiment, the nebulized liposomes, recovered in the three stages, were combined, purified and subsequently analyzed for the determination of NER%.

The higher phospholipid concentration led to a better packing of the RFP-loaded liposomal bilayers with a consequent higher stability of the three PC60 formulations during the nebulization process. The formulation ability to retain the drug was in the order: PC60 Chol OA (55% ± 1.4%) ≥ PC60 Chol (49% ± 7.9%) and PC60 (45% ± 3.1%). 

The size of nebulized vesicles showed a small increase in mean diameter and PI, probably because the nebulization process could have disrupted the liposomes, causing their fragmentation and reformation with different mean diameter and structure (data not shown).

**Table 3:** Nebulization Efficiency (NE%) and RFP retained into vesicles in the nebulized fraction (NER) of liposomes containing 30 or 60 mg/mL of PC.

		NE%	FPD (µg)	FPF (%)	NER%
PC30	Stage 1	35 ± 2.3	908 ± 23	9 ± 0.7	25 ± 0.7
	Stage 2	5 ± 0.5			
	Stage 3	4 ± 0.1			
PC30 Chol	Stage 1	48 ± 3.9	736 ± 12	7 ± 1.3	25 ± 1.0
	Stage 2	5 ± 0.8			
	Stage 3	3 ± 0.2			
PC30 Chol OA	Stage 1	51 ± 2.9	108 ± 32	11 ± 0.8	49 ± 3.3
	Stage 2	7 ± 0.2			
	Stage 3	4 ± 0.1			
PC60	Stage 1	41 ± 6.8	1300 ± 37	13 ± 0.9	45 ± 3.1
	Stage 2	10 ± 0.6			
	Stage 3	3 ± 0.1			
PC60 Chol	Stage 1	54 ± 3.7	1260 ± 29	13 ± 1.2	49 ± 7.9
	Stage 2	9 ± 0.2			
	Stage 3	3 ± 0.1			
PC60 Chol OA	Stage 1	64 ± 2.3	1685 ± 35	17 ± 1.7	55 ± 1.4
	Stage 2	12 ± 0.7			
	Stage 3	5 ± 0.1			

### 2.4. Cell Viability and Probe Uptake Studies

All the studied formulations showed very similar physico-chemical properties, but the three liposome formulations prepared with 60 mg/mL of binary mixture of P90H and P50 appeared more suitable for pulmonary delivery, thanks to their smaller size and higher homogeneous distribution, entrapment and nebulization efficiency. Taking into account these results, further studies were performed using only the three PC60 formulations.

**Figure 4:**
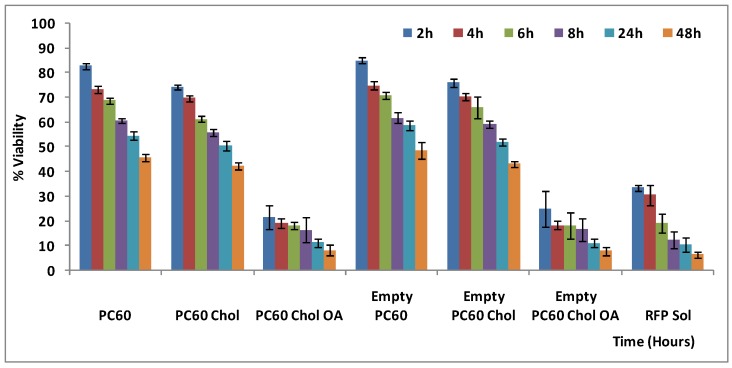
*In vitro* cytotoxic effect of empty and RFP-loaded liposomes compared with RFP solution on A549 cells at different incubation times.

*In vitro* toxicity and cell internalization studies of rifampicin liposome were carried out using alveolar epithelial lung cells of adenocarcinoma (A549). The toxicity was evaluated by the MTT cell viability test (Figure 4). PC60 and PC60 Chol showed low toxicity, and, at 4 h of incubation, the viability reached 70% for rifampicin loaded liposomes, and 80% for empty formulations. Successively, viability decreased gradually. At 24 h and 48 h of exposure, mortality increased up to 60%, compared to untreated control cells (100% viability). PC60 Chol OA liposomes showed important toxic effect, already evident after 2 and 4 h of incubation, to become more significant at 24 and 48 h, (mortality ~90%). Toxicity of rifampicin loaded liposomes was comparable to that of empty formulations, whereas rifampicin solution toxicity was much higher, reflecting the liposome ability to decrease the drug toxicity. However, in the presence of oleic acid (PC60 Chol OA), liposomes became toxic. Probably, fatty acids cause destabilization of membrane integrity, DNA fragmentation, and chromatin condensation, with consequent promotion of apoptosis and necrosis of the cells [34,35,36,37].

**Figure 5:**
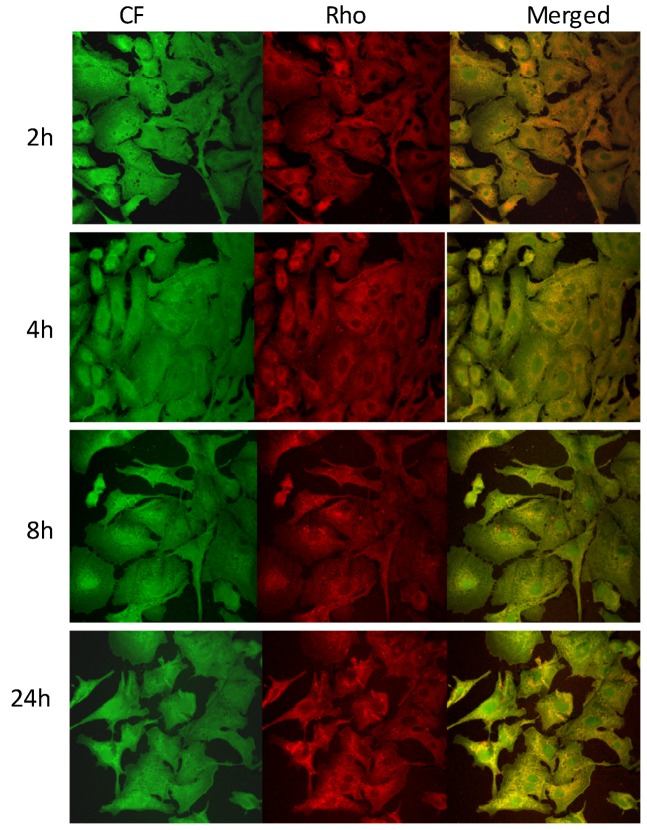
Images of A549 cells incubated for 2, 4, 8 and 24 h with Rho-PE labeled and CF loaded PC60 liposomes. The localization and intensity of dyes are displayed in red for Rho-PE, in green for CF.

**Figure 6:**
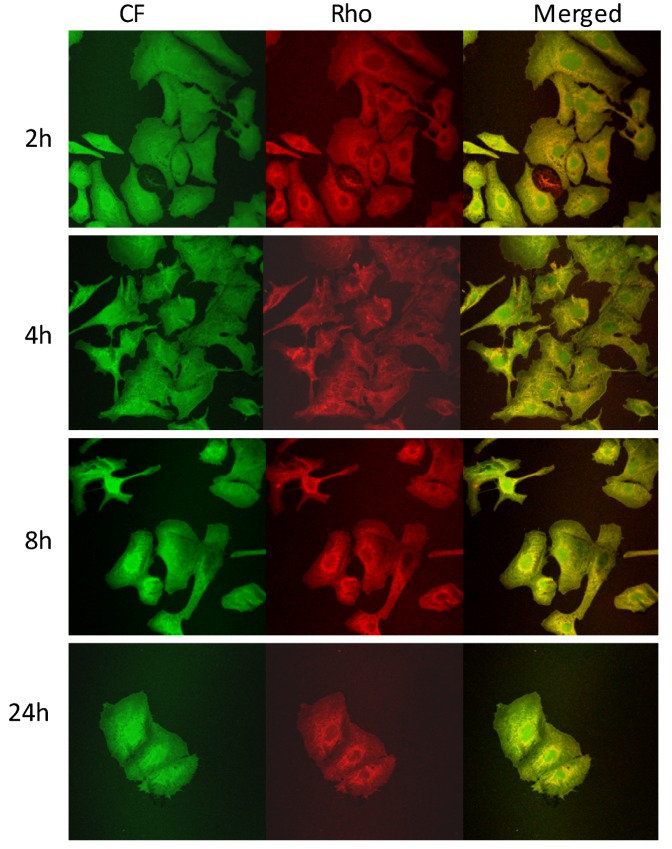
Images of A549 cells incubated for 2, 4, 8 and 24 h with Rho-PE labeled and CF loaded PC60 Chol OA liposomes. The localization and intensity of dyes are displayed in red for Rho-PE, in green for CF.

The ability of rifampicin-loaded liposomes to interact with A549 cells was evaluated using double fluorescently labeled vesicles, which were loaded with a membrane marker (Rho-PE) and a hydrophilic fluorescent probe (CF). CF was chosen as a marker of the liposome content, since it is a membrane impermeable probe especially used for investigating membrane integrity and permeability [38,39]. Cells were incubated with RFP-containing fluorescent liposomal formulations for 2, 4, 8 and 24 h and imaged by fluorescence microscopy to evaluate the marker distribution into the cells. An intense intracellular fluorescence was observed when cells were incubated with PC60 and PC60 Chol liposomes (Figure 5). Rho-PE (red) and CF (green) were localized throughout the cytoplasm and showed a good superposition (orange). Rho-PE was especially accumulated in the perinuclear area. Both probes were internalized in the A549 cells as confirmed by the appearance of an intracellular fluorescence that was diffuse, but weak, at 2 h of coincubation and became intense after 4 h of exposure. At 8 and 24 h, the marker fluorescence was intense and diffuse, but the number of cells decreased in agreement with the toxicity study. The decrease in the number of cells was more evident when PC60 Chol OA liposomes were used (Figure 6). In particular, at 8 and 24 h, the number of cells in the slides decreased, and cells appeared round and detached as a consequence of the toxic effect of oleic acid. 

In conclusion, cell uptake studies have highlighted the PC60 and PC60 Chol liposomes capability to interact with A549, thus facilitating drug internalization. Moreover, vesicular carrier has also been demonstrated to reduce the *in vitro* drug toxicity.

## 3. Experimental Section

### 3.1. Materials

Hydrogenated soy phosphatidylcholine (Phospholipon^®^ 90H, P90H) and soy phosphatidylcholine (Phospholipon^®^ 50, P50) were kindly supplied by AVG S.r.l. (Garbagnate Milanese, Milan, Italy) and Lipoid GmbH (Ludwigshafen, Germany). Phosphate buffer solution (PBS, pH 7.0) was purchased from Carlo Erba Reagents (Rodano, Italy). Rifampicin (RFP), cholesterol (Chol), oleic acid (OA) and all the other products were of analytical grade and were purchased from Sigma-Aldrich (Milan, Italy). The A549 epithelial alveolar cell line (passage 31) was a kind gift from Dr. Ben Forbes (School of Pharmacy, Kings College, London, United Kingdom).

### 3.2. Vesicle Preparation

Multilamellar vesicles (MLVs) were prepared according to the thin film hydration method with a slight modification (hydration in two steps) that allowed us to obtain more homogeneous vesicular populations [27]. Appropriate amounts of components, as reported in Table 1, were dissolved in chloroform, and subsequently, organic solvent was evaporated in order to obtain a thin film of lipids. The thin lipid film was hydrated in two steps working at 60 °C: at first, a part (50% of final volume) of dextrose (1 mM) in phosphate buffered saline solution (PBS, pH 7.0) was added under mechanical shaking, and the mixture was stirred for 1 h. Successively, the second aliquot of the hydrating medium was added, and the dispersion was shaken for another hour.

Sonicated vesicles (SUVs) were prepared by sonicating (5 s on and 2 s off) MLV dispersions at a temperature above the gel-liquid transition temperature, with a Soniprep 150 ultrasonic disintegrator (MSE Crowley, London, United Kingdom), until a clear opalescent dispersion was obtained. 

### 3.3. Vesicle Characterization

Morphology of liposomes was checked by a Jem1010, Jeol, transmission electron microscope (TEM). 

Size distribution (average diameter and polydispersity index, P.I.) of the samples was determined by Photon Correlation Spectroscopy (PCS) using a Zetasizer nano (Malvern Instrument, Worcestershire, United Kingdom). Before counting, the samples were diluted with PBS. Samples were backscattered by a helium–neon laser (633 nm) at an angle of 173° and at constant temperature of 25 °C.

Zeta potential was estimated using the Zetasizer nano by means of the M3-PALS (Phase Analysis Light Scattering) technique, which measures the particle electrophoretic mobility in a thermostated cell. All the samples were analyzed 24 h after their preparation.

Liposome dispersions were purified from the non-incorporated drug by exhaustive dialysis. Dispersions (1 mL) were dialyzed using a Spectra/Por^®^ membrane (12–14 kDa MW cut-off, 3 nm pore size; Spectrum Laboratories Inc., Rancho Dominguez, CA, United States) in PBS (1 L, pH 7.0) at room temperature for 2 h (by replacing PBS every 30 min), which were appropriate to allow the dissolution and consequent removal of the non-entrapped RFP (solubility pH 7.3: 2.5 mg/mL) and to avoid the destabilization of the vesicular suspension (e.g., osmotic swelling and vesicle fusion) as well. Incorporation efficiency (E%), expressed as the percentage of the encapsulated drug with respect to the amount of RFP initially used in liposomal preparation, was determined by UV Spectroscopy (UV) after disruption of vesicles. Vesicles were broken with 0.025% Triton X-100 in PBS. RFP content was quantified at 485 nm using a UV spectrophotometer.

### 3.4. Rheological Studies

Viscoelastic measurements were carried out using a Kinexus rotational rheometer (Malvern Instruments, UK) with data acquisition and elaboration software rSpace. All measurements were made in triplicate at 25 °C using a double-gap concentric cylinder DG25, useful for low-viscosity dispersions. Samples were allowed to rest for at least 300 s prior to analysis. Viscometry experiments were conducted in a shear range of 0.01–10 Pa. For frequency sweep tests, all samples were subjected to an initial amplitude sweep to determine the linear viscoelastic region (LVR) where the values of the moduli are independent of the applied deformation. Subsequent frequency sweep tests were performed from 0.01 to 10 Hz, and at a shear stress of 0.5 Pa. The oscillatory parameters used to compare the viscoelastic properties of the different dispersions were the storage modulus (G'), or elastic part of the response, and the loss modulus (G''), or viscous response. 

### 3.5. Nebulization Studies of Liposomes

As previously reported, liposome aerosols were generated by using an efficient high-output continuous-flow Markos Mefar MB2 air-jet nebulizer, driven by a Nebula compressor (Markos Mefar, Bovezzo, BS, Italy) operating at 7 L/min [21]. A volume of 3 mL of samples was used, and the aerosolized liposomes were collected in PBS using a modified three-stages glass impinger that contained 3 mL of PBS in the collecting flask. The aerosol was introduced into the device through a calibrated glass tube and a critical orifice delivering the aerosol jet 5 mm above the flask bottom. After aerosolization to dryness (10 min.), the impinger contents were collected, the impinger was washed with 2 mL of buffer, and samples were assayed to evaluate the effect of nebulization on liposomes and drug content. Total aerosol mass output (%) was determined by weighing the nebulizer before and after the nebulization of the different formulations. The total amount of nebulized formulation (collected into the apparatus) was determined. The nebulization efficiency (NE%) of microsphere formulations is defined as the total output of drug collected on the impinger as a percentage of the total amount of drug submitted to nebulization. Nebulized liposomes were then separated from the drug that was released from the vesicles during the process (by dyalisis, as described above), and finally, the retention of RFP in nebulized vesicles (NER%) was calculated as the ratio between aerolized and purified liposomes collected in flask and aerolized liposomes collected in flask × 100.

NER% = (aerolized and purified liposomes collected in flask)/(aerolized liposomes collected in flask) × 100.

### 3.6. Cell Cultures

Human A549 alveolar cells (at passage 85) were grown as confluent monolayers in 35 mm tissue culture dishes incubated in 100% humidity and 5% CO_2_ at 37 °C. Dulbecco’s Modified Eagle Medium: Nutrient Mixture F-12 (DMEM/F12) (Life Tecnologies Europe, Monza, Italy), supplemented with 10% heat-inactivated fetal bovine serum, 100 U/mL penicillin and 100 mg/mL streptomycin (Life Tecnologies Europe, Monza, Italy) was used as growth media. Confluent monolayer cells that form monolayers were harvested with trypsin (0.25%), centrifuged at low speed (1600*g*, 4 min), resuspended in fresh medium and plated at a concentration of 2 × 10^5^ cells/dish.

### 3.7. Cell Viability Studies (MTT Assay)

A549 cells were plated into 96-well plates at a density of 7.5 × 10^3^ cells/well. After 24 h, A549 cells were treated for 2, 4, 6, 8, 24 and 48 h with both empty and RFP-loaded liposomes and compared with RFP solution placed in the cells at the same concentration. The effect of on the viability of cells was determined by [3(4,5-dimethylthiazol-2-yl)-2,5-diphenyltetrazolium bromide] MTT assay [34]. The dye is reduced in mitochondria by succinic dehydrogenase to an insoluble violet formazan product. Briefly, 250 µL of MTT reagent (0.5 mg/mL in PBS) was added to each well, and after 2 h, the formed formazan crystals were dissolved in DMSO. The reaction was spectrophotometrically measured at 570 nm with a microplate reader (Synergy 4, ReaderBioTek Instruments, AHSI S.P.A, Bernareggio, Italy).

All experiments were repeated at least three times and in triplicates. Results are shown as percent of cell viability in comparison with non-treated control cells (100% viability).

### 3.8. Cellular Uptake of Rho-PE Labeled CF-Loaded Vesicles

The cell interactions and cellular uptake were investigated by confocal microscopy. For this purpose vesicles were labeled with a lipophilic fluorescent marker 1,2-dioleolyl-sn-glycero-3-phosphoethanolamine-*N*-(lissamine rhodamine B sulfonyl) (0.035 mg/mL; Rho-PE) and loaded with a hydrophilic fluorescent marker 5(6)-carboxyfluorescein (0.025 mg/mL; CF). The fluorescent probes were added during vesicle preparation in order to obtain Rho-PE labeled CF loaded vesicles, and fluorescent formulations were then purified from the non-entrapped markers by dialysis, as described above.

A549 cells were maintained in culture on glass slides of 30 mm diameter, and the experiments were performed when confluent monolayer was reached. Cells were incubated at 37 °C with Rho-PE labeled CF-loaded vesicles for 2, 4, 8 and 24 h. Before observations, cells were washed twice with DMEM/F12 to remove fluorescent vesicles and background fluorescence, fixed with a solution of 4% paraformaldehyde in PBS (pH 7.4) The images were obtained using a confocal microscope inverted FluoView FV1000 (Olympus, Barcelona, Spain) equipped with a laser to ultraviolet light/Visible; a 20× objective UPlanSApo was used. The Rho and CF were visualized respectively with a wavelength of excitation and emission of 559 nm and 578 nm and 470 nm and 535 nm.

### 3.9. Statistical Analysis of Data

Data analysis was carried out with the software package R, version 2.10.1. Results are expressed as the mean ± standard deviation. Multiple comparisons of means (Tukey test) were used to substantiate statistical differences between groups, while Student’s *t*-test was used for comparison between two samples. Significance was tested at the 0.05 level of probability (*p*).

## 4. Conclusions

In this work we prepared and characterized several rifampicin-loaded liposome formulations by using a binary mixture of hydrogenated soy phosphatidylcholine with a high transition temperature (52 °C) and soy phosphatidylcholine with a low transition temperature (~ −10 °C) at two lipid concentrations (30 and 60 mg/mL). Moreover, these two basic formulations (PC30 and PC 60) were modified by adding cholesterol alone or cholesterol and oleic acid.

Overall results obtained in this work show that PC60 liposomes, containing phospholipids at the higher concentration, appear the most suitable for nebulization and, among these formulations, PC60 and cholesterol liposomes are the best candidates as pulmonary delivery system for rifampicin. Indeed, this formulation showed a high encapsulation efficiency (~74%), a high nebulization efficiency (~66%) and a good capability of retaining the drug during the nebulization process (although there was low deposition in the 2° and 3° stages). The PC 60 formulation, made up with only phospholipids, while showing the highest encapsulation efficiency (~74%), was not able to be nebulized in high percentage due to the high viscosity. As for PC60 Chol OA liposomes, results obtained by characterization studies showed good encapsulation efficiency (~69%) and nebulization properties, but on the contrary, cell viability testing highlighted a toxic effect of the oleic acid. Finally, internalization studies showed the PC60 Chol ability to interact effectively with the A549 cells, favoring their uptake by the cells, while the MTT test demonstrated the liposome ability to reduce drug toxicity. 

These outcomes represent a base condition for further studies, in order to evaluate the aerodynamic behavior of the sprayed aqueous dispersions and to assess *in vivo* activity in infected animal models.
